# Altering gait variability with an ankle exoskeleton

**DOI:** 10.1371/journal.pone.0205088

**Published:** 2018-10-24

**Authors:** Prokopios Antonellis, Samuel Galle, Dirk De Clercq, Philippe Malcolm

**Affiliations:** 1 Department of Biomechanics and Center for Research in Human Movement Variability, University of Nebraska at Omaha, Omaha, Nebraska, United States of America; 2 Department of Movement and Sports Sciences, Ghent University, Ghent, Belgium; University of Colorado Boulder, UNITED STATES

## Abstract

Exoskeletons can influence human gait. A healthy gait is characterized by a certain amount of variability compared to a non-healthy gait that has more inherent variability; however which exoskeleton assistance parameters are necessary to avoid increasing gait variability or to potentially lower gait variability below that of unassisted walking are unknown. This study investigated the interaction effects of exoskeleton timing and power on gait variability. Ten healthy participants walked on a treadmill with bilateral ankle-foot exoskeletons under ten conditions with different timing (varied from 36% to 54% of the stride) and power (varied from 0.2 to 0.5 W∙kg^-1^) combinations. We used the largest Lyapunov exponent (LyE) and maximum Floquet multiplier (FM) to evaluate the stride-to-stride fluctuations of the kinematic time series. We found the lowest LyE at the ankle and a significant reduction versus powered-off with exoskeleton power (summed for both legs) of 0.45 W∙kg^-1^ and actuation timing at 48% of the stride cycle. At the knee, a significant positive effect of power and a negative interaction effect of power and timing were found for LyE. We found significant positive interaction effects of the square of timing and power for LyE at the knee and hip joints. In contrast, the FM at the ankle increased with increasing power and later timing. We found a significant negative effect of power and a positive interaction effect of power and timing for FM at the knee and no significant effects of any of the exoskeleton parameters for FM at the hip. The ability of the exoskeleton to reduce the LyE at the ankle joint offers new possibilities in terms of altering gait variability, which could have applications for using exoskeletons as rehabilitation devices. Further efforts could examine if it is possible to simultaneously reduce the LyE and FM at one or more lower limb joints.

## Introduction

Walking is a fundamental skill and the prime gait of humans. When we walk, our footprints (e.g., those noticed in the sand or snow) never repeat themselves exactly, demonstrating the step-to-step variability in a continuous cycle of movement. This variability is inherent within all biological systems. Variability can be defined as the variations that occur in motor performance across multiple repetitions of a task over time. Traditional linear measures such as standard deviation and coefficient of variation can be used to assess variability. Although these measures can indicate the magnitude of variability at certain time occurrences, the temporal evolution of movement patterns is ignored. Gait parameters are often treated algorithmically (i.e., smoothing, differentiation and normalization) to provide a mean picture of the individual’s movement that distorts the temporal structure of variability [1]. In contrast, measures from nonlinear dynamics examine how neuromuscular behavior changes over time and provide information about the organization or structure of gait [1,2].

A certain amount of variability is inherent in gait even in the absence of injury or pathology [3]. The subtle fluctuations that exist with healthy locomotion might indicate that the human locomotor system has maintained sufficient adaptability to facilitate maintaining an effective gait despite a continuously changing environment. An increase in gait variability renders the locomotor system more unstable and unpredictable [3–5]. Gait variability can quantify the stability of the human locomotor system. Increased gait variability has been associated with increased fall risk [6–13]. Based on nonlinear dynamics theory, highly variable kinematics indicate instability [8,14]. Indices, such as the maximum Floquet multiplier and largest Lyapunov exponent, are used for continuous joint or trunk kinematics to assess orbital and local stability, respectively. Both indices quantify the ability of the system to recover from small perturbations. For limit cycle systems with a constant fixed period, orbital stability is defined using the maximum Floquet multiplier, which quantifies the ability of a system’s state (from one gait cycle to the next) to return to the periodic limit cycle orbit after small perturbations [15]. The maximum Floquet multiplier was first used to assess the orbital stability of walking robots [15–21]. Human walking is not a strictly periodic movement. For such aperiodic systems, local stability is defined using the largest Lyapunov exponent, which quantifies the system’s state responses to very small perturbations occurring continuously in real time [15]. Largest Lyapunov exponent measurements have shown predictive validity with regard to falling in various observational and modeling studies [22]. The largest Lyapunov exponent was used to evaluate gait stability in different populations, including the elderly [13], patients with knee osteoarthritis [23], and amputees [24,25]. However, to the best of our knowledge, both the maximum Floquet multiplier and largest Lyapunov exponent indices have not been used to evaluate the effect of exoskeleton actuation parameters on gait variability.

Assistive wearable robotic devices, particularly exoskeletons, are becoming established technologies. Several lower limb exoskeletons have been developed that reduce the metabolic energy cost of walking [26–29] and hopping [30], increase walking speed [31], restore function in mobility-impaired people [32–34] and enhance performance during loaded walking [29,35]. To identify the effect of exoskeleton power magnitude on the metabolic cost of walking as well as the interaction with actuation timing, a recent study [36] of both actuation timing and exoskeleton power over a large range was conducted. Optimal actuation timing was achieved with an onset of 42% stride and an average power of 0.4 W∙kg^-1^, resulting in a 21% metabolic cost reduction compared to that of walking with the exoskeleton deactivated [36].

It is possible that exoskeletons, which are intended to make walking easier, may do so at the cost of increasing the variability of the locomotor system, thus making the locomotor system unstable, as was found in a study with a pneumatic ankle exoskeleton [21]. Conversely, it may be possible to develop exoskeletal assistance that reduces variability. This knowledge may be of interest to people that experience reduced balance and could benefit from exoskeleton assistance, such as individuals with peripheral arterial disease [4], chronic obstructive pulmonary disease [37], or anterior cruciate ligament deficiency [5] who demonstrate increased gait variability during walking.

The effects of exoskeleton power magnitude and interaction effects with actuation timing on gait variability within the human locomotor system are unknown. Studies on bipedal walking robots suggest that push-off before contralateral heel strike (i.e., early push-off) can lead to reduced stability [38,39] and thus greater gait variability. In human prosthesis walking, increased inter-stride variability in both push-off work and step length resulted from early push-off timing [40]. In contrast, longer double support times, which were found with later push-off actuation timing [40], increased the time during which both limbs can stabilize the body and thus reduced gait variability.

A first step in avoiding unwanted increased variability and potentially reducing variability with exoskeleton assistance is to examine the acute effect of exoskeleton walking in healthy individuals. Therefore, this study aimed to characterize the effect of ankle exoskeleton power and the interaction with actuation timing on gait variability. A tethered and powered plantar flexion-assisting exoskeleton was used to vary actuation onset timing and average exoskeleton power independently over a broad range to investigate the influence of these factors on gait variability. Based on findings with unilateral prostheses and bipedal walking robots [38,40], we hypothesized that late exoskeleton actuation timing would reduce gait variability. Based on a study that compared walking with and without exoskeleton assistance and found an increased maximum Floquet multiplier of ankle joint kinematics with exoskeleton assistance [21], we hypothesized that higher exoskeleton power would increase the maximum Floquet multiplier at the ankle. Finally, we hypothesized that the effect of actuation timing would be lower when actuation power and torque are lower and closer to the powered-off condition (i.e., exoskeleton power and torque equal zero). We expect the results of this study to guide follow-up experiments among individuals with limited mobility, eventually leading to the design of improved exoskeleton devices that can assist mobility without increasing gait variability during walking.

## Materials and methods

### Participants

We recruited ten healthy female participants (age 23 ± 1.2 years; body mass 61.0 ± 4.5 kg; and height 168.1 ± 5.2 cm). The sample size was chosen based on similar exoskeleton studies [26–29,41,42]. All participants had no previous experience with exoskeleton walking. This study was approved by the Ethical Committee of Ghent University Hospital, and all participants were required to provide written informed consent prior to the start of the study. Additional methodological details can be found in a previous study from our group [36].

### Exoskeleton

**Fig 1:**
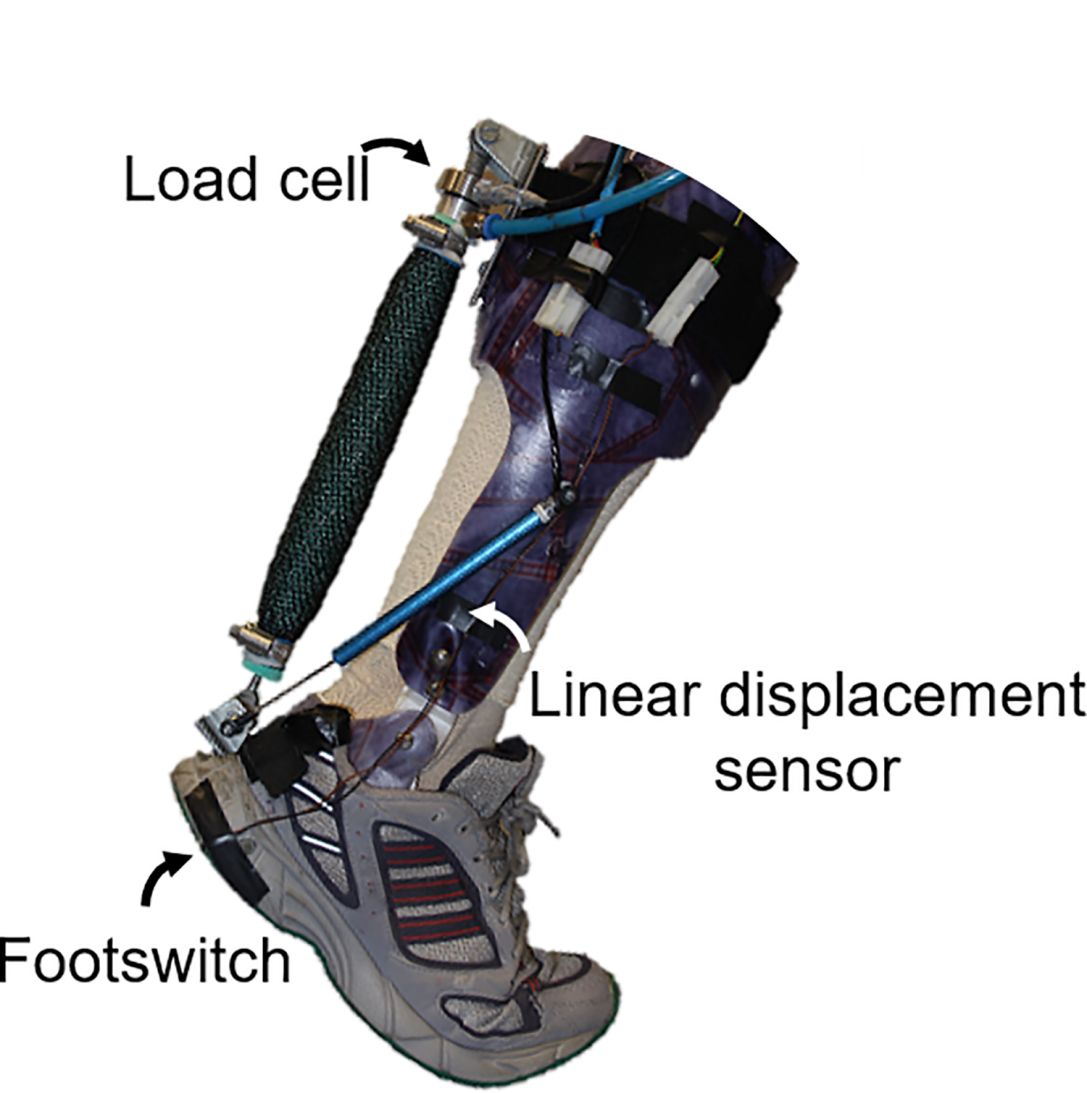
Exoskeleton and sensors. The exoskeleton has a load cell that measures the tension force of the pneumatic muscle, a linear displacement sensor that measures ankle kinematics, and a footswitch that is used to detect foot contact. The pneumatic muscles, when filled with compressed air, can shorten and can assist plantarflexion of the ankle during walking.

We used bilateral ankle exoskeletons powered with pneumatic muscles (Fig 1). Foot switches (Multimec 5E/5G, Mec, Ballerup, Denmark) were built in to detect foot contact. Load cells (100 Hz; 210 Series, Richmond Industries Ltd., Reading, United Kingdom) were connected between the exoskeleton and the pneumatic muscles to measure the pneumatic muscle force. Linear displacement sensors (100 Hz; SLS130, Penny & Giles, Christchurch, United Kingdom) were connected between the foot and shank sections of the exoskeletons to measure ankle joint angles [43]. More details on the exoskeleton design can be found in the Galle et al., [36] study.

### Experimental conditions

**Fig 2:**
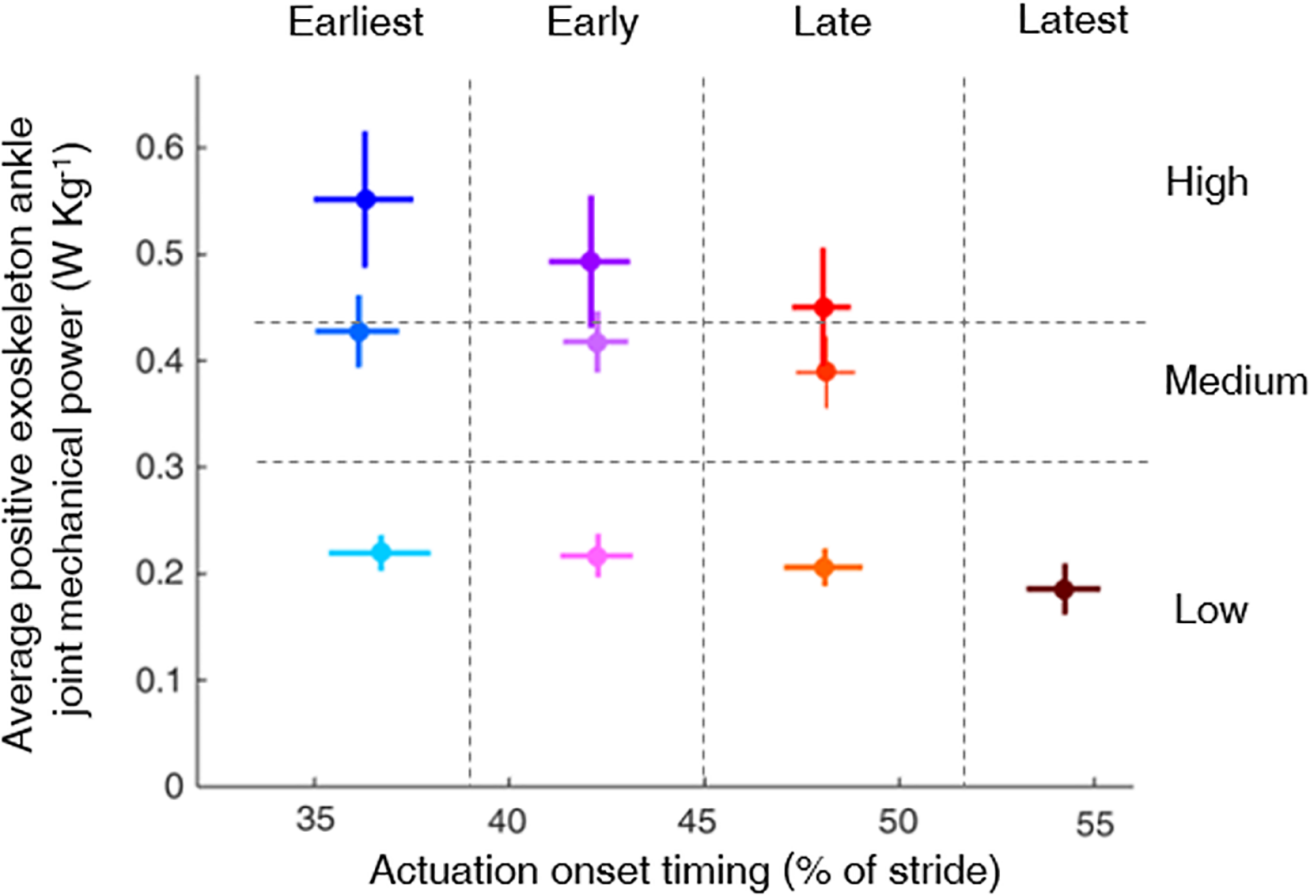
Parameter sweep of actuation timing and exoskeleton power. Actuation timing and exoskeleton power for the 10 powered exoskeleton conditions tested. Actuation onset timing is expressed as a percentage of the stride time and exoskeleton power is expressed as the average positive exoskeleton power over a stride, summed for both legs. The dots are population averages, and the lines are standard deviations. Each condition resulted in a distinct combination of timing and power. ([36], http://creativecommons.org/licenses/by/4.0/).

The experiment consisted of a habituation session and a data collection session with one week between sessions. We evaluated 12 conditions: a no-exoskeleton condition, in which participants walked without the exoskeleton; a powered-off condition, in which participants walked with the exoskeleton but without assistance from the pneumatic muscles; and 10 powered exoskeleton conditions. The range of timings was chosen to be centered around the timing that was previously found to minimize energy cost [27]. Moreover, the range of actuation magnitudes was chosen to go up to just beyond net biological ankle work [41,44,45] and exceed the largest range of previous ankle exoskeleton studies [46]. Due to bandwidth limitations of the pneumatic actuators, we were unable to use certain combinations of late timing and high actuation magnitude, and the power levels in the late timing conditions were more closely grouped than in the early timing conditions. A feedforward control algorithm in LabVIEW (National Instruments, Austin, TX, USA) [27,35,36,47] allowed exoskeleton programming to actuate with a specific desired onset timing and average positive power based on real-time measurements from the exoskeleton sensors. The 10 powered conditions were combinations of three actuation onset timings, 36 ± 1%, 42 ± 1% and 48 ± 1% of the stride (Earliest, Early and Late, respectively), for which three exoskeleton power levels were applied, 0.21 ± 0.02 W∙kg^-1^, 0.41 ± 0.03 W∙kg^-1^ and 0.50 ± 0.06 W∙kg^-1^ (Low, Medium and High, respectively), and a fourth actuation timing, 54 ± 1% of the stride (Latest) for which only one power level was applied (Low) [36] (Fig 2).

### Experimental protocol

The participants walked under all powered conditions, the powered-off condition and the no-exoskeleton condition on the treadmill at 1.25 m∙s^-1^. All conditions were applied at random and lasted four minutes with two minutes of rest between conditions.

### Data collection

**Fig 3:**
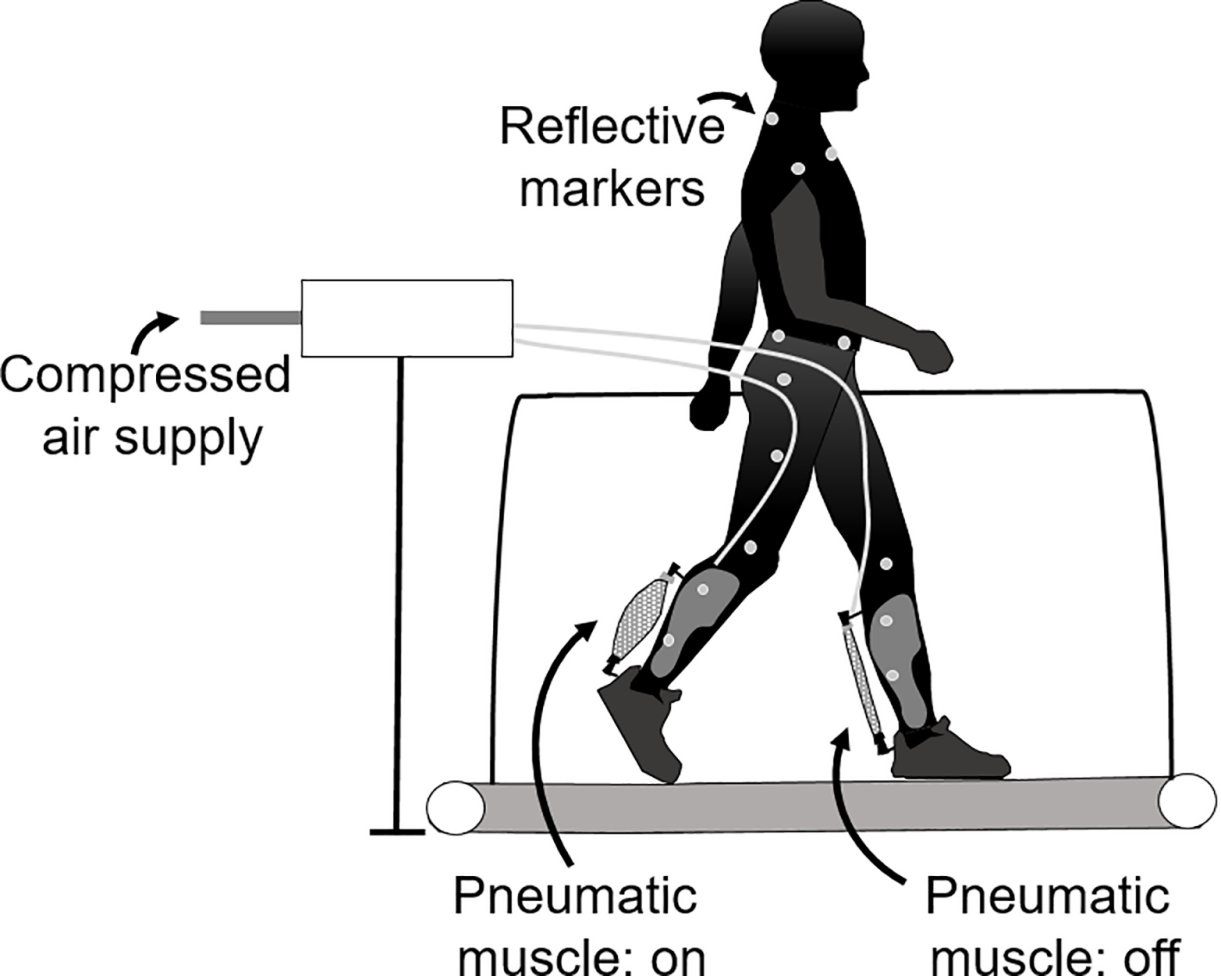
Experimental setup. Exoskeletons were worn on both legs. The pneumatic muscles were activated during push-off and assisted plantarflexion of the ankle during walking. Participants wore reflective markers for data collection.

Exoskeleton sensor data (foot switches, displacement sensors and load cells) were acquired continuously throughout the entire experiment (Fig 1). Full body 3D kinematics were recorded using 51 reflective markers (four per foot, two per exoskeleton foot segment, two per exoskeleton ankle joint, six per exoskeleton shank segment, two per knee joint, four on a plate connected to each thigh, six on the pelvis, and five on the torso) and 14 infrared cameras (200 Hz; Pro Reflex, Qualisys AB, Gothenburg, Sweden) with Qualisys software (Fig 3). Marker data were collected for 10 s during the third and last minute of each condition.

### Data analysis

Data were exported and processed in custom software using MATLAB (MathWorks Inc., Natick, MA, USA). All time series data were normalized to 100 frames from heel contact to the next heel contact using the spline interpolation function in MATLAB. All data from the left and right legs were averaged.

Kinematics were analyzed to evaluate the effects of exoskeleton assistance on walking patterns. An eight-segment model (two feet, two shanks, two thighs, one pelvis and one torso) was used to calculate sagittal plane joint angles with Visual 3D software (C-Motion, Germantown, MD, USA). Heel contact and toe-off were automatically detected using foot kinematics [48]. Toe-off timing was expressed as a percentage of the stride time.

Exoskeleton kinetics were calculated to assess actuation timing and exoskeleton power. Marker positions and pneumatic muscle force data were low-pass filtered using a fourth order Butterworth filter with a cut-off frequency of 12 Hz. We calculated the exoskeleton moment based on the load cell force and the moment arm of the pneumatic muscle. Actuation onset timing was calculated based on the maximum of the second derivative of the unfiltered exoskeleton torque to provide a robust measure of torque onset [40]. Average positive exoskeleton power was calculated as the numerical integration of positive instantaneous exoskeleton power over the stride divided by the stride time and summed for both legs.

The largest Lyapunov exponent (LyE) was used to quantify the mean rate of divergence of neighbored state space trajectories present in the attractor dynamics [49]. The LyE indicates the amount of variability in the system and is used as a walking balance metric [7,9,14,50]. Specifically, when referring to gait, LyE values provide a measure of the exponential divergence over time of the trajectories associated with each repetition of the stepping motion [51]. A LyE value would be zero when there is a perfectly repeating stepping pattern, a phenomenon that has not been observed in biological movement. Greater LyE values are associated with greater divergence across the trajectories of a movement as it is repeated. We used the LyE to investigate the variability in joint kinematic patterns, which are highly periodic [51] and because it considers the entire time series of the joint angle (it is not calculated from only one specific time point in each time series). More detailed explanations of the methods for calculating the LyE are outlined in previous studies [4,6,10,49]. All lower limb joint angle flexion/extension time series were cropped to 18 strides to compare a similar number of strides across participants. This number of strides is within the range of other studies that used between 6 and 30 strides [4,12,15,52]. The false nearest neighbor and average mutual information algorithms were used to calculate the embedding dimension and time lag for each time series, respectively [4,53,54]. The number of time points to propagate before finding a new nearest neighbor was set equal to 3 [51,54]. The maximum angle (from the reference trajectory in which the new nearest neighbor must reside) was set to 0.3 radians [49,51,54]. The minimum scale length was set to 0.0001 (minimum distance to selection of the new nearest neighbor [49,51,54]). Finally, the maximum scale length was set to 0.1 times the maximum length of the attractor (maximum distance to selection of the new nearest neighbor [49,51,54]).

The maximum Floquet multiplier (FM) was used to quantify the average divergence rate of state space trajectories in response to small disturbances. While the LyE can be analyzed for all trajectories of a system, the FM is used for closed trajectories (i.e., periodic orbits) in the state space. Therefore, the FM assumes that gait is a periodic motion and that the analyzed gait variable demonstrates such limit cycle behavior (e.g., joint motion but not stride times or stride lengths) [22]. Greater FM values are associated with slower convergence/faster divergence across movement trajectories, indicating poorer stability within the system. Calculating the FM requires that the state is sampled at a discrete event in the walking cycle [15,18]. According to previous studies on steady state walking, the FM is insensitive to the choice of Poincare section [15,18]. Previous studies on the use of FM analysis have sampled the Poincare sections at heel strikes as they represent biologically meaningful events within the gait cycle [19–21]. Furthermore, we sampled the state at the left and right heel strikes forming two Poincare sections [21]. Twenty heel strikes were recorded during every condition for each side. States for each Poincare section were created into two matrices, and a least squares estimate of a linear fit was used to estimate the Jacobian of the Poincare map (Jp) [18]. The FM was determined by the Jp eigenvalues. The method for calculating the FM is described in previous studies [15,18,21,22].

### Statistical analysis

For each condition, the means and standard deviations of the average LyE and average FM were calculated across participants. To determine the effects and interaction effects of exoskeleton actuation timing and power across all conditions, we performed a mixed-model ANOVA with subject number as random effect, different terms based on average positive exoskeleton power and timing as fixed effects, and variability parameters as dependent parameter. For each parameter, we started by evaluating a formula with the following terms using mixed-model ANOVA:

$${\mathrm{Variability}\;\mathrm{parameter}=\mathrm{Intercept}+b\cdot \mathrm{Power}+c\cdot \mathrm{Power}}^{2}+\left({d\cdot \mathrm{Timing}+e\cdot \mathrm{Timing}}^{2}\right)\cdot \mathrm{Power}$$ (1)

Whereby Power is average bilateral exoskeleton positive power expressed in W∙kg^-1^ and Timing is onset timing expressed in percent of stride time (i.e., on a scale from 0 to 100).

Since we did not have prior knowledge on whether the effect of average positive exoskeleton power on variability metrics will show a monotonously rising or descending or U-shaped or inverted U-shaped relationship, the part of the formula with b · Power + c · Power^2^ allows the surface fit to follow these different possible relationships. Similarly, since we have no prior knowledge on the effect of actuation timing on variability parameters, the part of the formula with d · Timing + e · Timing^2^ allows the surface fit to follow different possible relationships. However, we do expect that the effect of Timing must become gradually smaller and ultimately non-existent when approaching powered-off. The part of the formula with d · Timing + e · Timing^2^ is multiplied by Power to reflect this expected behavior. Starting from this initial form, we have used backward stepwise elimination to adapt the equation for each variability metric by removing terms that do not significantly contribute. Our statistical method (mixed-model ANOVA and elimination of terms that do not significantly contribute) is similar to methods used in [55]. Based on the mixed-model ANOVA, we generated contour plots showing each variability metric versus exoskeleton power and timing. To estimate the region between the exoskeleton conditions with low power and the powered-off condition, we also included data from the powered-off condition as input to the mixed-model ANOVA. Since timing cannot be defined in the powered-off condition and since we expect that the effect of timing becomes non-existent when approaching powered-off, we used the variability value of the powered-off condition together with power equal to zero and timing equal to each of the four timing settings as input data for the mixed-model ANOVA. By using this approach, the variability at powered-off is plotted as a line with a constant color scale for variability on the bottom of each contour plot similar to our previous study [36]. Finally, pairwise comparisons were performed using paired t-tests with a Šidák-Holm correction for multiple comparisons to search for differences between the powered exoskeleton conditions and the powered-off condition, between the powered exoskeleton conditions and the no-exoskeleton condition, and between the powered-off exoskeleton condition and the no-exoskeleton condition. We used a significance threshold of 0.05 for both the mixed-model ANOVA and the pairwise comparisons. All statistical analyses were performed in MATLAB.

## Results

### Largest lyapunov exponent

**Fig 4:**
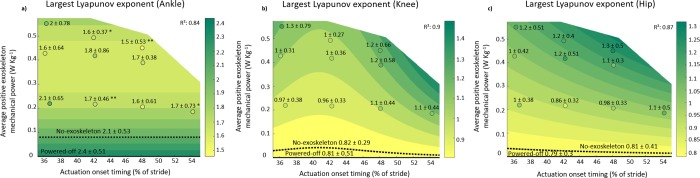
Largest Lyapunov exponent of lower limb joint angles is depicted versus actuation timing and average positive exoskeleton power. a) Ankle, b) knee and c) hip. The contour plot indicates the result of the mixed-model ANOVA. Numbers show the population mean ± s.d. for each condition. * indicates a statistically significant reduction versus powered-off based on paired t-tests with Šidák-Holm correction (P ≤ 0.05). ** indicates a statistically significant reduction versus powered-off based on paired t-tests with Šidák-Holm correction (P ≤ 0.01). The black dotted line represents the intersection of the contour plot with the LyE value of the no-exoskeleton condition.

At the ankle, Power and Power^2^ significantly affected the LyE (P_Power_ < 0.001; P_Power_^2^ = 0.001, ANOVA; Table 1; Fig 4A). We found the lowest LyE in the Late-High condition (1.5 ± 0.53), in which the average power was 0.45 ± 0.05 W∙kg^-1^. The LyE in this condition was 0.65 lower than in the no-exoskeleton condition, but this difference was not significant (P = 0.153, t-test). The highest LyE occurred in the powered-off condition (2.4 ± 0.51). The LyE in the Early-Low, Early-High, Late-High and Latest-Low powered conditions were significantly lower (P-values of 0.005, 0.014, 0.001, and 0.026, respectively, t-tests) than that in the powered-off condition (Fig 4A). There were no significant differences between the powered-off and the no-exoskeleton condition (P = 0.471, t-test). None of the powered conditions were significantly different compared to the no-exoskeleton condition (P-values ≥ 0.153, t-tests).

**Table 1:** Results of mixed-model ANOVA (random effect: Participant; fixed effects: Combinations of exoskeleton power and actuation timing; outcome parameters: LyE and FM of the ankle, knee and hip joints). Values indicate significant resulting equation coefficients.

Variables	Intercept Coefficient	Power	Power^2^	Power·Timing	Power·Timing^2^
Ankle LyE	2.4**	-4.1**	5.5**	NA	NA
Knee LyE	0.8**	14*	NA	-0.66*	0.0081*
Hip LyE	0.79**	NA	NA	NA	0.0004**
Ankle FM	0.18**	NA	NA	0.0044**	NA
Knee FM	0.25**	-0.5*	NA	0.0116*	NA
Hip FM	0.27**	NA	NA	NA	NA

*P ≤ 0.05

**P ≤ 0.01; NA = not applicable to final model due to non-significant contribution. The coefficients are for multiplication with power values in W∙kg^-1^ and timing values in % of stride (i.e., from 0 to 100).

At the knee, Power significantly affected the LyE (P_Power_ = 0.014, ANOVA; Table 1; Fig 4B). There were significant interaction effects of Power·Timing as well as of the Power·Timing^2^ on the LyE (P_Power · Timing_ = 0.015; P_Power · Timing_^2^ = 0.011, ANOVA; Table 1; Fig 4B). We found the lowest LyE in the powered-off condition (0.81 ± 0.51) and the highest LyE in the Earliest-High condition (1.3 ± 0.79). There were no significant differences between the powered-off and the no-exoskeleton condition (P = 0.910, t-test). None of the powered conditions were significantly different compared to the powered-off condition (P-values ≥ 0.255, t-tests) or compared to the no-exoskeleton condition (P-values ≥ 0.123, t-tests).

At the hip, there was a significant interaction effect of the Power·Timing^2^ on the LyE (P_Power · Timing_^2^ < 0.001, ANOVA; Table 1; Fig 4C). We found the lowest LyE in the powered-off condition (0.79 ± 0.3) and the highest LyE in the Late-High condition (1.3 ± 0.5). There were no significant differences between the powered-off and the no-exoskeleton condition (P = 0.890, t-test). None of the powered conditions were significantly different compared to the powered-off condition (P-values ≥ 0.132, t-tests) or compared to the no-exoskeleton condition (P-values ≥ 0.271, t-tests).

### Maximum Floquet multiplier

**Fig 5:**
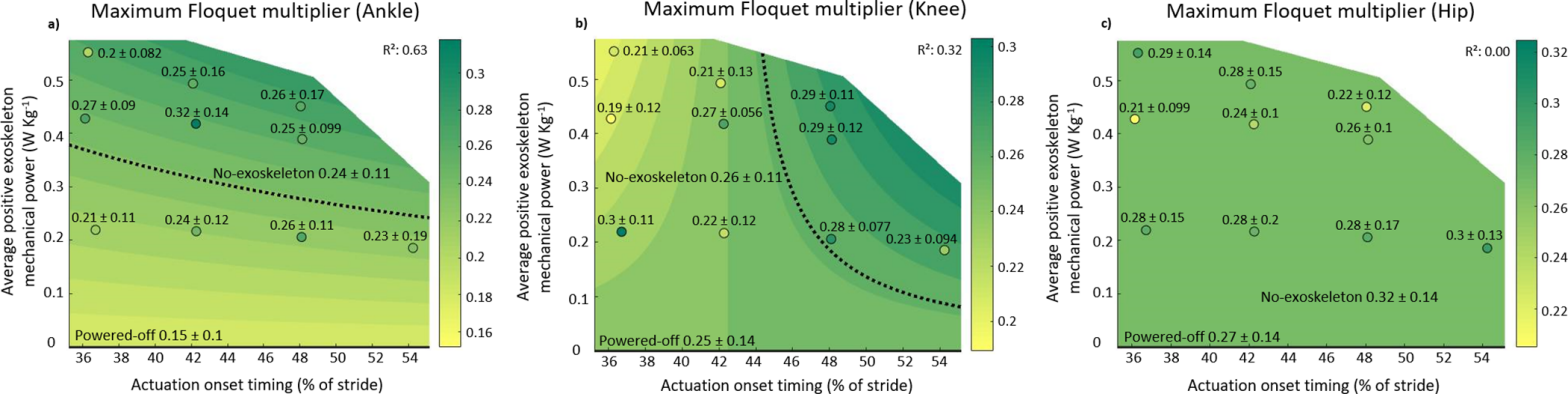
Maximum Floquet multiplier of lower limb joint angles is depicted versus actuation timing and average positive exoskeleton power. a) Ankle, b) knee, and c) hip. The contour plot indicates the result of mixed-model ANOVA. Numbers show the population mean ± s.d. for each condition. The black dotted line represents the intersection of the contour plot with the FM value of the no-exoskeleton condition.

At the ankle, there were significant interaction effects of Power·Timing on the FM (P_Power ·Timing_ < 0.001, ANOVA; Table 1, Fig 5A). We found the lowest FM in the powered-off condition (0.15 ± 0.1) and the highest FM in the Early-Medium condition (0.32 ± 0.14). There were no significant differences between the powered-off and the no-exoskeleton condition (P = 0.791, t-test). None of the powered conditions were significantly different compared to the powered-off condition (P-values ≥ 0.340, t-tests) or compared to the no-exoskeleton condition (P-values ≥ 0.877, t-tests).

At the knee, Power significantly affected the FM (P_Power_ = 0.038, ANOVA; Table 1; Fig 5B). There were significant interaction effects of Power·Timing on the FM (P_Power · Timing_ = 0.047, ANOVA; Table 1; Fig 5B). We found the lowest FM in the Earliest-Medium condition (0.19 ± 0.12) and the highest FM in the Late-High condition (0.29 ± 0.11). There were no significant differences between the powered-off and the no-exoskeleton condition (P = 0.994, t-test). None of the powered conditions were significantly different compared to the powered-off condition (P-values ≥ 0.999, t-tests) or compared to the no-exoskeleton condition (P-values ≥ 0.955, t-tests).

At the hip, there were no interaction effects of Power·Timing or the Power·Timing^2^ on the FM (P_Power · Timing_ ≥ 0.05; P_Power · Timing_^2^ ≥ 0.05, ANOVA; Table 1; Fig 5C). We found the lowest FM in the Earliest-Medium condition (0.21 ± 0.09) and the highest FM in the no-exoskeleton condition (0.32 ± 0.14). There were no significant differences between the powered-off and the no-exoskeleton condition (P = 0.974, t-test). None of the powered conditions were significantly different compared to the powered-off condition (P-values ≥ 0.999, t-tests) or compared to the no-exoskeleton condition (P-values ≥ 0.471, t-tests).

## Discussion

This study investigated the influence of exoskeleton power and the interaction with timing on gait variability. We found that actuation from an ankle exoskeleton can alter gait dynamics. The LyE showed that increased exoskeleton power reduced gait variability at the ankle compared to powered-off, but the FM showed the opposite effect. More specific, the Late-High condition with bilateral exoskeleton power of 0.45 W∙kg^-1^ and actuation timing at 48% resulted in the lowest LyE of all powered conditions and a lower LyE than walking with the exoskeleton without assistance from the pneumatic muscles (powered-off). Conversely, the FM showed that higher exoskeleton power and late timing increased gait variability at the ankle, and the FM was highest with bilateral exoskeleton power of 0.37 W∙kg^-1^ and actuation timing of 42%. At the knee, we found that the LyE was lowest in the powered-off condition, and the Early-High condition resulted in the highest LyE. No effects of actuation timing or power were found at the hip for the LyE or at the hip and knee for the FM.

The LyE at the ankle showed that higher levels of exoskeleton power resulted in greater reductions in gait variability. As such, according to the LyE at the ankle it appears that the ankle kinematics of walking with the exoskeleton powered are more deterministic than walking with the exoskeleton powered-off. Deterministic is defined as less randomness in the development of future states of the human locomotor system. Deterministic behavior of the neuromuscular system allows humans to adapt gait to changing environmental conditions. For example, predictable aspects of the behavior of our locomotor system allow us to devise strategies for walking on uneven terrain or walking around obstacles [4]. On the other hand, the FM at the ankle showed that higher exoskeleton power resulted in increases in gait variability. This result in FM indicates that the higher levels of exoskeleton power decreased the ability of the neuromuscular system to return toward a fixed periodic motion.

This raises the question of why there are different trends in gait variability according to the LyE and FM at the ankle joint. Local stability (i.e., LyE) and orbital stability (i.e., FM) metrics quantify different properties of system dynamics [15]. Local dynamic stability is used to measure the divergence in terms of space and time variables. It quantifies the average exponential rates of divergence of neighboring trajectories in state space [10]. In contrast, orbital dynamic stability measures the tendency of the dynamic system to diverge (or converge) back to its trajectory in a discrete manner only in space. To calculate the LyE in our study, we used data from the entire time series of each joint angle including both active and inactive periods of the exoskeleton. The FM, however, was calculated at a single point during the cycle (i.e., heel strike). This time point is approximately in the middle of the active period of the contralateral exoskeleton, which could be the reason why FM is affected by exoskeleton power. Of note, FM assumes that the locomotor system is strictly periodic, but it remains uncertain whether human gait is periodic since stride cycles differ in length and this variability has a non-random structure [22]. In contrast to FM, the calculation of LyE takes into account the variations between strides and the specific trajectories the human locomotor system follows through state space from one stride to the next. This includes both phases where the exoskeleton is active and phases where the exoskeleton is inactive, which could explain the different trend found for the LyE compared to FM.

Actuation onset and exoskeleton power significantly affected the LyE and FM at the knee, and the LyE at the hip. However, we found no effects on FM at the hip. These findings might be because our exoskeleton device was designed to assist ankle joint movement, and thus, more dynamic degrees of freedom must be controlled for the hip, which need increased control [51,56]. Future research is needed to determine appropriate exoskeleton designs along with effective actuation timings and exoskeleton power to reduce gait variability at the knee and hip joints, possibly combined with experiments on incline and decline walking set-ups that require increased biological work at the knee and hip. Likely, the goal is not to reduce variability to the absolute achievable minimum at each joint because that could take away the freedom of movement that the wearer needs to respond to small perturbations (e.g., surface irregularities).

The exoskeleton power profile applied at each push-off was not fully controlled and exoskeleton power might have varied between each stride. This could explain the increases in gait variability that we found at the different joints. Increases in gait variability may contribute to increased fall rates and mobility problems. This is reflected in findings for the healthy young and elderly [7], healthy elderly and elderly fallers [10,11], and healthy individuals and patients with Parkinson's and Huntington's disease [57], which have been linked to increased falling risk and decreased physical function. However, a relationship between variability and falling has not been demonstrated in exoskeleton walking.

At this time, only one previous study from Norris et al., [21] has used the LyE and the FM to compare the differences in gait variability between wearing the exoskeleton with and without push-off power assistance. In that study, the authors also assessed the metabolic energy cost during walking with the exoskeleton with and without power assistance. They found that the FM increased when the exoskeleton was powered, but the metabolic energy cost was reduced. Additionally, they found no changes in the LyE between wearing the exoskeleton with and without assistance. Similar to their study, we found that the FM increased at the ankle, but surprisingly, the LyE was reduced. Possible explanations could be that in their study the LyE and the FM were calculated from the sagittal plane angular velocities and accelerations of the foot and shank sections of the exoskeleton [21]. The authors employed a different exoskeleton control algorithm that augmented ankle power at push-off based on detection of foot-flat [21]. Their algorithm may vary more in detecting foot-flat leading to increased gait variability during powered exoskeleton walking. Lastly, they used a different exoskeleton design and did not evaluate multiple assistance parameter settings (actuation timing, assistance magnitude, etc.).

We previously studied the effect of actuation timing and average exoskeleton power on metabolic energy cost and found that the highest reduction in metabolic cost occurred with actuation timing at 42% of the stride and an average exoskeleton power of 0.42 W∙kg^−1^ [36]. In the present study, the LyE at the ankle was lower at the powered condition with actuation timing at 48% of the stride and an average exoskeleton power of 0.45 W∙kg^−1^. On the other hand, the FM at the ankle increased in all powered conditions with the highest amount present at the powered condition with actuation timing at 42% of the stride and an average exoskeleton power of 0.41 W∙kg^-1^. Future research could examine the relationship between gait variability and metabolic energy consumption during powered exoskeleton walking.

In our study, no significant differences were found in gait variability for both LyE and FM at the ankle, knee and hip joints between powered-off exoskeleton walking and walking without the exoskeleton. These results suggest that the weight (i.e., added load) of the exoskeleton did not alter gait variability. A previous study on load carrying found that load carriage up to 30% of body weight did not have an effect on joint kinematic variability [58]. We were only able to reduce the LyE during powered exoskeleton walking compared to powered-off at the (ankle) joint that was directly assisted by the exoskeleton, since LyE was the highest in the powered-off condition at the ankle and the lowest in the powered-off condition at the knee and hip joints. It might also be possible to reduce the LyE at the knee and hip joints with knee and hip exoskeletons, or perhaps it could even be possible to reduce the LyE simultaneously at multiple joints by assisting multiple joints together with a multi-joint exoskeleton. Moreover, we demonstrated significant reductions in the LyE at the ankle compared to that in the powered-off condition, but we found significant increases in the FM at the ankle with higher exoskeleton power. We also found no statistically significant differences between the powered exoskeleton conditions and the no-exoskeleton condition for both LyE and FM metrics. Therefore, at this stage, our exoskeleton cannot be used as a tool to reduce gait variability below the level of walking without an exoskeleton but can be used as a device to lower the metabolic cost of walking [36] possibly without increasing gait variability compared to that of walking without the exoskeleton.

The results of our study may not extend to global (or total) stability in which the response of the human system to larger perturbations (e.g., tripping or slipping) is examined [15,59]. Global stability can be assessed by measuring the attraction of the locomotor system [60], or by detecting the largest perturbations that the locomotor system could tolerate without falling over [61]. These analyses need a large number of large perturbations to be applied to the system to examine its reaction [15]. Therefore, they can be used to assess the stability of robots or simulations, but it would be difficult to conduct this type of analyses in human studies.

One possible application of the present study could be the further development of exoskeleton actuation patterns for mobility assistance that do not reduce metabolic cost at the expense of increasing gait variability. Our current study shows that this might be possible since none of the conditions significantly increased gait variability compared to walking without the exoskeleton, and an optimal actuation pattern was previously shown to reduce metabolic cost below the level of walking without the exoskeleton [36]. Our findings provide descriptive data of gait variability values during walking with an exoskeleton in healthy participants that could be used as hypothetical target values for walking with exoskeletons in other (impaired) populations. Another possible application could be the development of actuation patterns for exercise therapy for reducing gait variability in populations characterized by increased gait variability. We believe that our findings are of interest since they show that it is possible to alter certain nonlinear gait variability metrics. Further research with advanced optimization methods [42,62–64] including larger sample sizes is needed to investigate if similar results can be obtained in patient populations.

The present study has the following limitations. Every participant responded differently to exoskeleton assistance. We investigated the average effects of actuation timing and exoskeleton power on variability metrics across the population; however, the actuation parameter settings that increased or reduced variability metrics seemed to differ between participants. Additionally, participants might have adapted differently if the exoskeleton walking trials were longer than four minutes. By using individual optimization methods, it may be possible to further reduce gait variability. Moreover, there is no data in the literature about what are the ideal variability values for different metrics (i.e., LyE and FM) during walking. We also do not know what the ideal amount of variability is for walking with an exoskeleton, but we believe that it could be possible to influence certain variability parameters using exoskeletons in clinical populations that have increased gait variability. Furthermore, overground and treadmill walking differ biomechanically and physiologically [65,66]. Treadmills could limit fluctuations in walking that are normally present in overground walking. Contradictory results on the differences in gait variability between treadmill and overground walking exist in the literature [52,67,68]. Thus, the treadmill may have affected the variability of gait data. Lastly, we could not fully control the shape of the exoskeleton power curve, and it is possible that other actuation parameters affect gait variability to a greater extent. For example, our exoskeleton testbed could not deliver sufficient power to elaborate the latest onset timing thoroughly. It would be useful to explore more exoskeleton power with late timing. A future approach could be to evaluate the effect of other actuation profile parameters on the exoskeleton walking variability.

## Conclusions

This study examined the effects of ankle exoskeleton power and the interaction with actuation timing on gait variability. During exoskeleton walking with pneumatic muscle assistance, individuals ambulate with reduced LyE at the ankle joint, which is one measure of gait variability. We found the lowest LyE for the ankle with actuation timing at 48% of the stride and average exoskeleton power at approximately 0.45 W∙kg^-1^. No statistically significant differences in gait variability were reported at the ankle between the powered exoskeleton conditions and walking without the exoskeleton. On the other hand, the FM indicated that higher exoskeleton power increased gait variability at the ankle, with a bilateral exoskeleton power of 0.37 W∙kg^-1^ corresponding to the highest FM. Additionally, exoskeleton power and actuation timing had effects on the LyE and FM at the knee, and the LyE at the hip. In contrast, exoskeleton power and actuation timing did not significantly affect the FM at the hip. Our study results provide new insights into the human response to external assistance in terms of altering and possibly reducing gait variability, which may benefit future studies focused on designing and controlling lower limb exoskeleton devices. This needs to be further tested for the FM via experiments possibly using diverse and pathological populations. Future work should investigate whether this approach provides similar changes and potentially even improvements in patient populations with reduced exercise tolerance, and examine the sensitivity and specificity of the two nonlinear methods used in this study to aid the design of improved exoskeleton devices for rehabilitation and clinical use.

## Supporting information

S1 File Data summary. (XLSX)

## References

[1] Stergiou N. Innovative analyses of human movement. Human Kinetics; 2004.

[2] Sosnoff JJ, Valantine AD, Newell KM. Independence between the amount and structure of variability at low force levels. Neurosci Lett. 2006;392: 165–169. 10.1016/j.neulet.2005.09.010 16188384

[3] Stergiou N, Harbourne RT, Cavanaugh JT. Optimal Movement Variability: A New Theoretical Perspective for Neurologic Physical Therapy. J Neurol Phys Ther. 2006;30: 120–129. 10.1097/01.NPT.0000281949.48193.d9 17029655

[4] Myers SA, Johanning JM, Stergiou N, Celis RI, Robinson L, Pipinos II. Gait variability is altered in patients with peripheral arterial disease. J Vasc Surg. The Society for Vascular Surgery; 2009;49: 924–931.e1. 10.1016/j.jvs.2008.11.020 19217749

[5] Stergiou N, Moraiti C, Giakas G, Ristanis S, Georgoulis AD. The effect of the walking speed on the stability of the anterior cruciate ligament deficient knee. Clin Biomech. 2004;19: 957–963. 10.1016/j.clinbiomech.2004.06.008 15475129

[6] Bruijn SM, Bregman DJJ, Meijer OG, Beek PJ, van Dieën JH. Maximum Lyapunov exponents as predictors of global gait stability: A modelling approach. Med Eng Phys. Institute of Physics and Engineering in Medicine; 2012;34: 428–436. 10.1016/j.medengphy.2011.07.024 21856204

[7] Buzzi UH, Stergiou N, Kurz MJ, Hageman PA, Heidel J. Nonlinear dynamics indicates aging affects variability during gait. Clin Biomech. 2003;18: 435–443. 10.1016/S0268-0033(03)00029-9 12763440

[8] Hausdorff JM, Rios DA, Edelberg HK. Gait variability and fall risk in community-living older adults: A 1-year prospective study. Arch Phys Med Rehabil. 2001;82: 1050–1056. 10.1053/apmr.2001.24893 11494184

[9] Kurz MJ, Markopoulou K, Stergiou N. Attractor divergence as a metric for assessing walking balance. Nonlinear Dynamics Psychol Life Sci. 2010;14: 151–164. 20346260

[10] Lockhart TE Te, Liu J. Differentiating fall-prone and healthy adults using local dynamic stability. Ergonomics. 2008;51: 1860–1872. 10.1080/00140130802567079 19034782 PMC2892176

[11] Maki BE. Gait Changes in Older Adults: Predictors of Falls or Indicators of Fear? J Am Geriatr Soc. 1997;45: 313–320. 10.1111/j.1532-5415.1997.tb00946 9063277

[12] van Schooten KS, Sloot LH, Bruijn SM, Kingma H, Meijer OG, Pijnappels M, et al Sensitivity of trunk variability and stability measures to balance impairments induced by galvanic vestibular stimulation during gait. Gait Posture. 2011;33: 656–660. 10.1016/j.gaitpost.2011.02.017 21435878

[13] Toebes MJP, Hoozemans MJM, Furrer R, Dekker J, Van Dieën JH. Local dynamic stability and variability of gait are associated with fall history in elderly subjects. Gait Posture. 2012;36: 527–531. 10.1016/j.gaitpost.2012.05.016 22748312

[14] England SA, Granata KP. The influence of gait speed on local dynamic stability of walking. Gait Posture. 2007;25: 172–178. 10.1016/j.gaitpost.2006.03.003 16621565 PMC1785331

[15] Dingwell JB, Hyun Gu Kang. Differences Between Local and Orbital Dynamic Stability During Human Walking. J Biomech Eng. 2007;129: 586 10.1115/1.2746383 17655480

[16] Kuo A. D. Stabilization of Lateral Motion in Passive Dynamic Walking. Int J Rob Res. 1999;18: 917–930. 10.1177/02783649922066655

[17] Russell S, Granata KP, Sheth P. Virtual slope control of a forward dynamic bipedal walker. J Biomech Eng. 2005;127: 114–122. 10.1115/1.1835358 15868794 PMC1633712

[18] Hurmuzlu Y, Basdogan C. On the Measurement of Dynamic Stability of Human Locomotion. Journal of Biomechanical Engineering-Transactions of the Asme. 1994 pp. 30–36. 10.1115/1.2895701 8189711

[19] Arellano CJ, O’Connor DP, Layne C, Kurz MJ. The independent effect of added mass on the stability of the sagittal plane leg kinematics during steady-state human walking. J Exp Biol. 2009;212: 1965–1970. 10.1242/jeb.026153 19483014

[20] Granata KP, Lockhart TE. Dynamic stability differences in fall prone and healthy adults. J Electromyogr Kinesiol. 2008;18: 172–178. 10.1016/j.jelekin.2007.06.008 17686633 PMC2895268

[21] Norris JA, Marsh AP, Granata KP, Ross SD. Positive Feedback in Powered Exoskeletons: Improved Metabolic Efficiency at the Cost of Reduced Stability? Vol 5 6th Int Conf Multibody Syst Nonlinear Dyn Control Parts A, B, C. 2007; 1619–1626. 10.1115/DETC2007-35657

[22] Bruijn SM, Meijer OG, Beek PJ, van Dieën JH. Assessing the stability of human locomotion: a review of current measures. J R Soc Interface. 2013;10: 20120999 10.1098/rsif.2012.0999 23516062 PMC3645408

[23] Fallah Yakhdani HR, Bafghi HA, Meijer OG, Bruijn SM, Dikkenberg N van den, Stibbe AB, et al Stability and variability of knee kinematics during gait in knee osteoarthritis before and after replacement surgery. Clin Biomech. 2010;25: 230–236. 10.1016/j.clinbiomech.2009.12.003 20060628

[24] Lamoth CJC, Ainsworth E, Polomski W, Houdijk H. Variability and stability analysis of walking of transfemoral amputees. Med Eng Phys. Institute of Physics and Engineering in Medicine; 2010;32: 1009–1014. 10.1016/j.medengphy.2010.07.001 20685147

[25] Segal AD, Orendurff MS, Czerniecki JM, Shofer JB, Klute GK. Local dynamic stability of amputees wearing a torsion adapter compared to a rigid adapter during straight-line and turning gait. J Biomech. 2010;43: 2798–2803. 10.1016/j.jbiomech.2010.05.038 20719315

[26] Collins SH, Wiggin MB, Sawicki GS. Reducing the energy cost of human walking using an unpowered exoskeleton. Nature. 2015;522: 212–215. 10.1038/nature14288 25830889 PMC4481882

[27] Malcolm P, Derave W, Galle S, De Clercq D. A Simple Exoskeleton That Assists Plantarflexion Can Reduce the Metabolic Cost of Human Walking. PLoS One. 2013;8 10.1371/journal.pone.0056137 23418524 PMC3571952

[28] Mooney LM, Rouse EJ, Herr HM, Patterson M, Roberts W, Lau W, et al Autonomous exoskeleton reduces metabolic cost of human walking during load carriage. J Neuroeng Rehabil. 2014;11: 80 10.1186/1743-0003-11-80 24885527 PMC4036406

[29] Panizzolo FA, Galiana I, Asbeck AT, Siviy C, Schmidt K, Holt KG, et al A biologically-inspired multi-joint soft exosuit that can reduce the energy cost of loaded walking. J Neuroeng Rehabil. Journal of NeuroEngineering and Rehabilitation; 2016;13: 43 10.1186/s12984-016-0150-9 27169361 PMC4864923

[30] Grabowski AM, Herr HM. Leg exoskeleton reduces the metabolic cost of human hopping. J Appl Physiol. 2009;107: 670–678. 10.1152/japplphysiol.91609.2008 19423835

[31] Norris JA, Granata KP, Mitros MR, Byrne EM, Marsh AP. Effect of augmented plantarflexion power on preferred walking speed and economy in young and older adults. Gait Posture. 2007;25: 620–627. 10.1016/j.gaitpost.2006.07.002 16905320

[32] Awad LN, Bae J, O’Donnell K, De Rossi SMM, Hendron K, Sloot LH, et al A soft robotic exosuit improves walking in patients after stroke. Sci Transl Med. 2017;9: eaai9084 10.1126/scitranslmed.aai9084 28747517

[33] Takahashi KZ, Lewek MD, Sawicki GS. A neuromechanics-based powered ankle exoskeleton to assist walking post-stroke: a feasibility study. J Neuroeng Rehabil. 2015;12: 23 10.1186/s12984-015-0015-7 25889283 PMC4367918

[34] Veneman JF, Kruidhof R, Hekman EEG, Ekkelenkamp R, Van Asseldonk EHF, Van Der Kooij H. Design and evaluation of the LOPES exoskeleton robot for interactive gait rehabilitation. IEEE Trans Neural Syst Rehabil Eng. 2007;15: 379–386. 10.1109/TNSRE.2003.818185 17894270

[35] Galle S, Malcolm P, Derave W, De Clercq D. Enhancing performance during inclined loaded walking with a powered ankle-foot exoskeleton. Eur J Appl Physiol. 2014;114: 2341–2351. 10.1007/s00421-014-2955-1 25064193

[36] Galle S, Malcolm P, Collins SH, De Clercq D. Reducing the metabolic cost of walking with an ankle exoskeleton: interaction between actuation timing and power. J Neuroeng Rehabil. Journal of NeuroEngineering and Rehabilitation; 2017;14: 35 10.1186/s12984-017-0235-0 28449684 PMC5408443

[37] Yentes JM, Rennard SI, Schmid KK, Blanke D, Stergiou N. Patients with chronic obstructive pulmonary disease walk with altered step time and step width variability as compared with healthy control subjects. Ann Am Thorac Soc. 2017;14: 858–866. 10.1513/AnnalsATS.201607-547OC 28267374 PMC5566305

[38] Bhounsule PA, Cortell J, Grewal A, Hendriksen B, Karssen JGD, Paul C, et al Low-bandwidth reflex-based control for lower power walking: 65 km on a single battery charge. Int J Rob Res. 2014;33: 1305–1321.

[39] Collins SH, Ruina A. A bipedal walking robot with efficient and human-like gait. Proc—IEEE Int Conf Robot Autom. 2005;2005: 1983–1988. 10.1109/ROBOT.2005.1570404

[40] Malcolm P, Quesada RE, Caputo JM, Collins SH. The influence of push-off timing in a robotic ankle-foot prosthesis on the energetics and mechanics of walking. J Neuroeng Rehabil. 2015;12: 21 10.1186/s12984-015-0014-8 25889201 PMC4404655

[41] Sawicki GS, Ferris DP. Mechanics and energetics of level walking with powered ankle exoskeletons. J Exp Biol. 2008;211: 1402–1413. 10.1242/jeb.009241 18424674

[42] Ding Y, Kim M, Kuindersma S, Walsh CJ. Human-in-the-loop optimization of hip assistance with a soft exosuit during walking. Sci Robot. 2018;5438: 1–9. 10.1126/scirobotics.aar5438 33141683

[43] Galle S, Derave W, Bossuyt F, Calders P, Malcolm P, De Clercq D. Exoskeleton plantarflexion assistance for elderly. Gait Posture. 2017;52: 183–188. 10.1016/j.gaitpost.2016.11.040 27915222

[44] Mooney LM, Herr HM. Biomechanical walking mechanisms underlying the metabolic reduction caused by an autonomous exoskeleton. J Neuroeng Rehabil. Journal of NeuroEngineering and Rehabilitation; 2016;13: 4 10.1186/s12984-016-0111-3 26817449 PMC4730720

[45] Sawicki GS, Ferris DP. Powered ankle exoskeletons reveal the metabolic cost of plantar flexor mechanical work during walking with longer steps at constant step frequency. J Exp Biol. 2009;212: 21–31. 10.1242/jeb.017269 19088207

[46] Quinlivan BT, Lee S, Malcolm P, Rossi DM, Grimmer M, Siviy C, et al Assistance magnitude versus metabolic cost reductions for a tethered multiarticular soft exosuit. Sci Robot. 2017;2: eaah4416 10.1126/scirobotics.aah4416 33157865

[47] Galle S, Malcolm P, Derave W, De Clercq D. Adaptation to walking with an exoskeleton that assists ankle extension. Gait Posture. 2013;38: 495–499. 10.1016/j.gaitpost.2013.01.029 23465319

[48] O’Connor CM, Thorpe SK, O’Malley MJ, Vaughan CL. Automatic detection of gait events using kinematic data. Gait Posture. 2007;25: 469–474. 10.1016/j.gaitpost.2006.05.016 16876414

[49] Wolf A, Swift J, Swinney H, Vastano J. Determining Lyapunov exponents from a time series. Phys D Nonlinear Phenom. 1985; 285–317.

[50] Kurz MJ, Stergiou N. An artificial neural network that utilizes hip joint actuations to control bifurcations and chaos in a passive dynamic bipedal walking model. Biol Cybern. 2005;93: 213–221. 10.1007/s00422-005-0579-6 16059784

[51] Wurdeman SR, Myers SA, Jacobsen AL, Stergiou N. Adaptation and prosthesis effects on stride-to-stride fluctuations in amputee gait. PLoS One. 2014;9 10.1371/journal.pone.0100125 24956384 PMC4067312

[52] Dingwell JB, Cusumano JP, Cavanagh PR, Sternad D. Local Dynamic Stability Versus Kinematic Variability of Continuous Overground and Treadmill Walking. J Biomech Eng. 2001;123: 27 10.1115/1.1336798 11277298

[53] Abarbanel HDI. Analysis of observed chaotic data. New York, NY, USA: Springer-Verlag; 1996.

[54] Wurdeman SR, Myers SA, Stergiou N. Transtibial amputee joint motion has increased attractor divergence during walking compared to non-amputee gait. Ann Biomed Eng. 2013;41: 806–813. 10.1007/s10439-012-0705-2 23180032 PMC3596479

[55] Liew BXW, Morris S, Netto K. The effects of load carriage on joint work at different running velocities. J Biomech. 2016;49: 3275–3280. 10.1016/j.jbiomech.2016.08.012 27567569

[56] Newell KM, Broderick M, Deutsch K, Slifkin A. Task goals and change in dynamical degrees of freedom with motor learning. J Exp Psychol Hum Percept Perform. 2003;29: 379–387. 12760622 10.1037/0096-1523.29.2.379

[57] Hausdorff JM. Gait dynamics, fractals and falls: Finding meaning in the stride-to-stride fluctuations of human walking. Hum Mov Sci. 2007;26: 555–589. 10.1016/j.humov.2007.05.003 17618701 PMC2267927

[58] Arellano CJ, Layne CS, O’Connor DP, Scott-Pandorf M, Kurz MJ. Does load carrying influence sagittal plane locomotive stability? Med Sci Sports Exerc. 2009;41: 620–627. 10.1249/MSS.0b013e31818a0ea4 19204588

[59] Pavol MJ, Owings TM, Foley KT, Grabiner MD. Mechanisms leading to a fall from an induced trip in healthy older adults. J Gerontol A Biol Sci Med Sci. 2001;56: M428–M437. 10.1093/gerona/56.7.M428 11445602

[60] Wisse M, Schwab AL, van der Linde RQ, van der Helm FCT. How to keep from falling forward: Elementary swing leg action for passive dynamic walkers. IEEE Trans Robot. 2005;21: 393–401. 10.1109/TRO.2004.838030

[61] Mombaur KD, Longman RW, Bock HG, Schlöder JP. Open-loop stable running. Robotica. Cambridge University Press; 2005;23: 21–33. 10.1017/S026357470400058X

[62] Kim M, Ding Y, Malcolm P, Speeckaert J, Siviy CJ, Walsh CJ, et al Human-in-the-loop Bayesian optimization of wearable device parameters. PLoS One. 2017;12 10.1371/journal.pone.0184054 28926613 PMC5604949

[63] Felt W, Selinger JC, Donelan JM, Remy CD. “Body-In-The-Loop”: Optimizing Device Parameters Using Measures of Instantaneous Energetic Cost. PLoS One. 2015;10: e0135342 10.1371/journal.pone.0135342 26288361 PMC4545892

[64] Zhang J, Fiers P, Witte KA, Jackson RW, Poggensee KL, Atkeson CG, et al Human-in-the-loop optimization of exoskeleton assistance during walking. Science (80). 2017;356: 1280–1283. 10.1126/science.aal5054 28642437

[65] Lee SJ, Hidler J. Biomechanics of overground vs. treadmill walking in healthy individuals. J Appl Physiol. 2008;104: 747–755. 10.1152/japplphysiol.01380.2006 18048582

[66] Parvataneni K, Ploeg L, Olney SJ, Brouwer B. Kinematic, kinetic and metabolic parameters of treadmill versus overground walking in healthy older adults. Clin Biomech. 2009;24: 95–100. 10.1016/j.clinbiomech.2008.07.002 18976839

[67] Ojeda L V., Rebula JR, Kuo AD, Adamczyk PG. Influence of contextual task constraints on preferred stride parameters and their variabilities during human walking. Med Eng Phys. 2015;37: 929–936. 10.1016/j.medengphy.2015.06.010 26250066 PMC4604025

[68] Terrier P, Dériaz O. Kinematic variability, fractal dynamics and local dynamic stability of treadmill walking. J Neuroeng Rehabil. 2011;8: 12 10.1186/1743-0003-8-12 21345241 PMC3060113

